# Can Computer Tomography Predict Compromise of Cardiac Structures After Percutaneous Closure of Interatrial Septal Defects?

**DOI:** 10.4021/cr109w

**Published:** 2011-11-20

**Authors:** Philipp Wagdi

**Affiliations:** a Interventional Cardiology, HerzZentrum Hirslanden, Witellikerstr. 36, 8008 Zurich, Switzerland

**Keywords:** ASD, PFO, Percutaneous closure, Complications, Computer tomography

## Abstract

**Background:**

Erosion of a cardiac structure after device closure of an interatrial septal communication (IASC-C), although rare, is a major and severe adverse event which may be underreported. On the other hand, unexplained episodes of transient chest pain occur more often and may be quite distressing. We sought to define the parameters relating the devices to the adjacent cardiac structures and to determine whether computer tomography (CT) could predict erosion of atrial or aortic wall or precordial pain symptoms occurring in the first months after device implantation.

**Methods:**

Retrospective observational study of 20 patients who underwent CT for de novo chest pain occurring after IASC-C or as a diagnostic test for suspected or proven coronary artery disease (CAD). Clinical follow up was for 20.5 ± 17.6 (6-84) months. CT was done 18 ± 10 (2-28) weeks after IASC-C.

**Results:**

Indentation of the aortic root was found in 11 (55%) patients, the left atrial wall in 13 (65%) and the right atrial wall in eight (40%) of patients. Contact without indentation was found in nine (45%), 6 (30%) and 11 (55%) of patients respectively.

**Conclusions:**

Device indenting of the left and right atrial, as well as the aortic wall, occured in the majority of the patients examined after IASC-C. This finding may explain bouts of chest pain after the intervention in some patients, but does not predict clinically relevant erosion of a cardiac structure.

## Introduction

**Figure 1:**
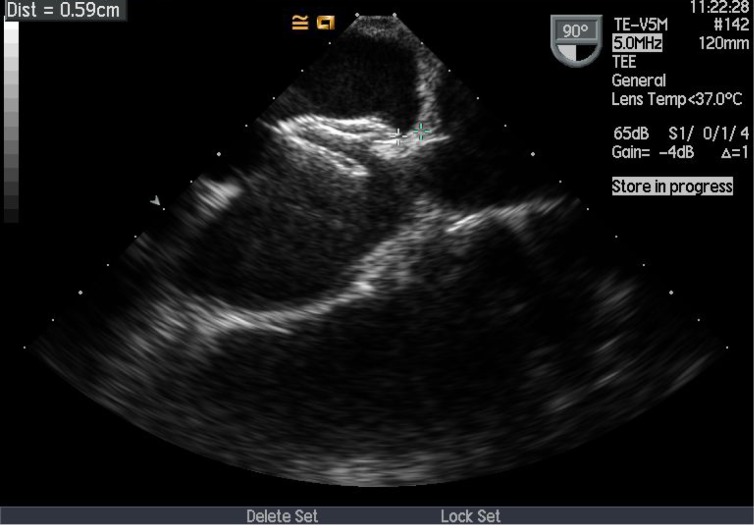
Echocardiographic documentation that prior to release, no device impingement of the atrial roof by the 20 mm Amplatzer™ device.

Erosion of a cardiac structure after percutaneous closure of an interatrial septal communication (IASC) is a rare but dramatic event [1]. It obviously confronts the physician with the question of how the event could have been avoided. Larger series of high volume centres [2, 3] report an adverse event incidence that may be related to erosion of 0.8% (5/641) and 0.4% (2/525). The etiology of “cardiac tamponade and/or pericardiocentesis” is not discussed in detail therein [2, 3]. To date this adverse event has occurred in one out of 122 patients (0.8 %) in our (single operator) series. To try to prevent a spatial conflict between the device and the surrounding cardiac structures, we routinely balloon size the defect using both fluoroscopy and echocardiography and determine the distance between the aortic rim, the atrial walls and the catheter lying across the patent foramen ovale (PFO) or atrial septal defect (ASD). Before releasing the chosen device, we then ask the Echocardiographer whether the respective device arms are on the correct side and whether any impingement of cardiac structures is seen. Mainly concerned are the aortic root, the atrial roof and floor: if the device lies flush to the structure, we usually deploy it (Fig. 1). However, if the device is seen to be indenting a structure, then it is be retrieved and, if possible, a smaller size or different device used. Despite proceeding this way, one of our patients experienced device perforation and tamponade seven months after closure of an ASD [1]. As reported previously, impingement of the device had been excluded by transesophageal echocardiography (TEE) during the procedure [1]. The patient experienced a severe bout of chest pain two weeks after implantation, a TEE and a computer tomography (CT) were immediately performed and ruled out tamponade, wall erosion, coronary artery disease (CAD), aortic dissection or pulmonary embolism. The patient had then remained asymptomatic for seven months, till the adverse event occurred.

We sought to determine whether CT could point out the patients at risk, and whether there was a correlation between the size of the device implanted and that of the atria on one hand, and the degree of impingement of the device on cardiac structures on the other. As documented [1], the intrainterventional TEE had not detected impingement of cardiac structures in this patient, which is the reason why we decided to review our CT (and not TEE) data for the investigation of this question.

## Methods

We retrospectively reviewed the data of twenty patients in whom an IASC-C had been performed and who later underwent CT for a clinical indication, not as a routine follow up after the intervention. Clinical characteristics are shown in Table 1. In ten patients, CT was indicated for precordial chest pain (we do not routinely perform coronary angiography before IASC-C in asymptomatic patients). The chest pain was severe and acute in seven of the patients and occurred 18 weeks (± 10; 2-28) after IASC-C. In five asymptomatic patients, CT was indicated because of pathological stress test findings during routine follow up. Four patients with documented CAD underwent CT for follow up. In one patient CT was indicated to rule out pulmonary embolism or neoplasm. We have recently demonstrated the reliability of CT in the diagnosis of coronary artery disease in our respective institutions [4]. Chest pain occurred 14.6 ± 11 (1-28) weeks after device implantation. All CT scans were performed on a second generation dual-source CT scanner. Depending on the heart rate, patients were scanned in the high-pitch mode (heart rate < 62 bpm) or in the step-and-shoot mode (heart rate > 62 bpm). The average effective radiation dose was 3.1 ± 1.2 (0.5-4.4) milliSievert. Contrast medium dose was 77 ± 6.1 (65-90) mL. No adverse event occurred in relation to CT.

**Table 1:** Clinical Charateristics of Patients (n = 20) After IASC-C

Age in years	Gender	FU	BMI
56.3 ± 8.4 (38-70)	16 females	20.5 ± 17.6 (6-84)	26.5 ± 6.1 (19.5-42.6)

BMI: body mass index; FU: followup in months after IASC-C; IASC-C: interatrial septal defect closure.

The following parameters were determined: long and short axis dimensions of both atria, minimal distance between the device and the aorta, the atrial roof and floor, protrusion of device into superior vena cava (if applicable). Additionally, presence of a rest-shunt was reported.

The following measurements were defined in the four chamber view: Right atrial short axis: from the middle of the septum perpendicularly to the lateral right atrial wall. Right atrial long axis: from the origin of tricuspid annulus perpendicularly to the right atrial posterior wall. Left atrial short axis: from the middle of the septum perpendicularly to the lateral left atrial wall. Left atrial long axis: from the origin of mitral annulus perpendicularly to the left atrial posterior wall.

**Figure 2:**
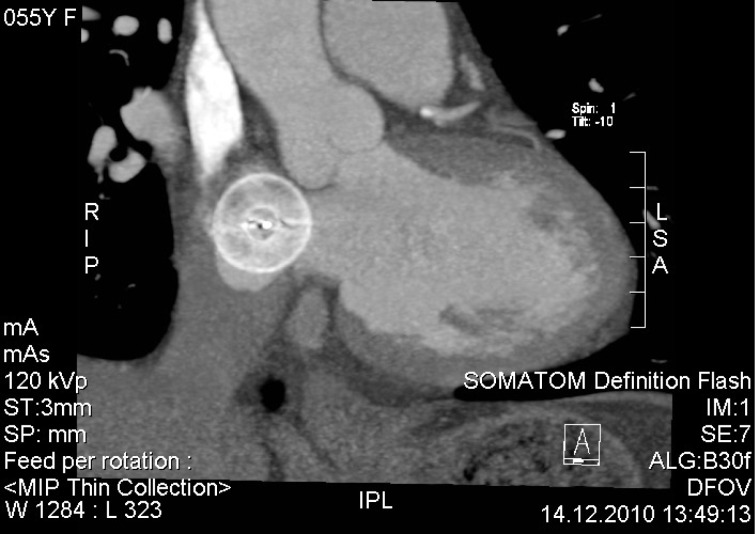
Example of a device in contact but not impinging the aortic wall.

**Figure 3:**
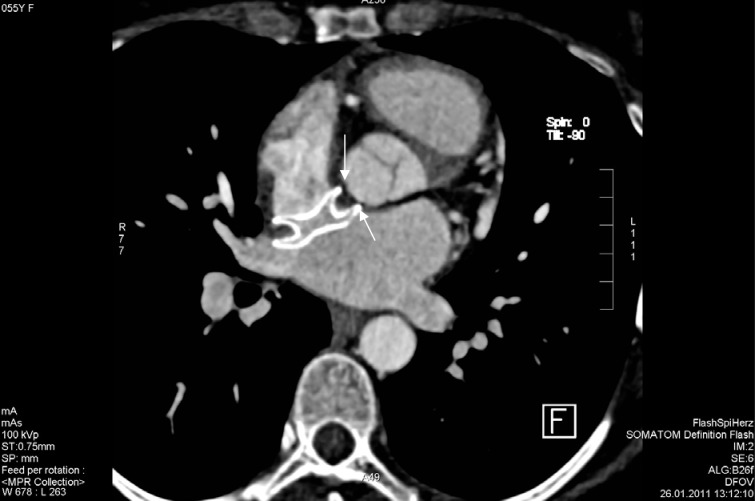
Device seen indenting the aortic root wall (arrows) by a maximum of 4 mm as computed from the magnified image.

The relation of the device to the cardiac structures was defined as the minimal measurable distance between the device and the respective atrial or aortic root structure. A device edge barely in contact but not impinging the structure was assigned zero mm (Fig. 2). Impingement of an atrial or aortic root area was measured in mm and assigned a positive value (Fig. 3). If the device was not in contact with a cardiac structure a negative value was assigned. In addition, the maximal protrusion in mm of a device into the lumen of the superior vena cava was measured.

All data were analysed using commercially available statistical software (StatView 5.0; ASA Institute Inc., Cary, NC, USA).

## Results

**Figure 4:**
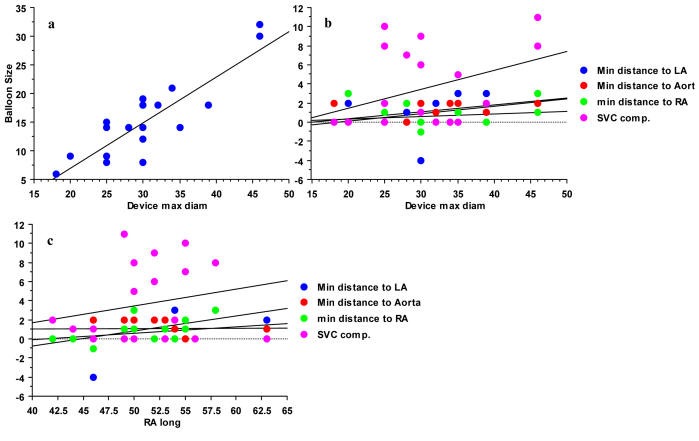
(a) Correlation between device size and preimplantation balloon measurement; r^2^ = 0.735. In 6 patients a larger device than would be expected was used because of a large septal aneurysma and/or a cribriform defect. (b) Lack of correlation between device size in millimetres (mm) and variables of compromission. Smallest measurable distance of device edge to left (LA) or right (RA) atrial wall or aorta in mm. Device protrusion into superior vena cava (SVC) in mm. All correlation coefficients r^2^ < 0.3. (c) Correlation between long axis dimensions of the right atrium in millimetres (mm) and variables of compromission. Smallest measurable distance of device edge to left (LA) or right (RA) atrial wall or aorta in mm. Device protrusion into SVC in mm. Only protrusion into superior vena cava (SVC) correlated moderately with the long axis of the RA (r^2^ = 0.53).

The IASC consisted of an atrial septal defect in five patients and of a patent foramen ovale (PFO) in 15 patients, four of which presented with a large aneurysm and two with a cribriform defect. Balloon sizing revealed a defect of 15.4 ± 6.9 (6-32) mm. The devices were 12 Amplatzer™, four Atriasept™, three Figulla™, one Solysafe™. Apart from information concerning the IASC closure, patency of a previously implanted stent in the left anterior descending coronary artery (LAD) was confirmed, and in two patients a 60% LAD stenosis was diagnosed. In all other patients CAD was excluded, as well as a pulmonary pathology in one of the patients. In all patients, except the one in whom a late perforation occurred [1], the symptoms subsided and the course was uneventful. Table 2 resumes the measurements obtained for the cardiac structures under examination. In all patients the device was either in contact or indenting the aortic root, in the majority it was indenting one of the atrial walls (Table 3). Not unexpectedly, there was a fair correlation between balloon sizing of the defect and the device size implanted (Fig. 4). No correlation was found between device size or atrial dimensions on the one hand, and device encroachement on or proximity to aortic and atrial walls (Fig. 4, Table 3) on the other hand. Maximal device diameter was: one device each of 18, 20, 28, 32 and 34 mm, five devices each of 25 and 30 mm, three devices of 35 mm, two devices of 46 mm. Left atrial long axis diameter measured < 48 mm in seven patients, 49-60 mm in 11 and > 60 mm in two patients. Left atrial short axis diameter was < 40 mm in 17 patients, 40-46 mm in three. Right atrial long axis dimensions were below 50 mm in 11 patients, 50-60 mm in eight patients and 63 mm in one patient. Right atrial short axis measured less than 41 mm in three patients, 42-50 mm in four, 51-60 mm in 11 and > 61 mm in two patients. Not unexpectedly, the smallest devices (18 and 20 mm), showed the least potential for encroachement. In one patient the device indented only the right atrial wall by 2 mm, in the other both right (3 mm) and left (2 mm) walls were indented.

**Table 2:** Dimensions Measured

Max DS	LA long	LA short	RA long	RA short
30.4 ± 7.4 (18-46)	51.6 ± 6 (44-65)	34 ± 5.5 (24-46)	50.8 ± 5.1 (42-63)	50.6 ± 8 (36-64)

DS: device size in mm (= maximal diameter of the largest disc as indicated by the manufacturer); LA: left atrium; Long: long axis dimension in mm; RA: right atrium; Short: short axis dimension in mm.

**Table 3:** Number of Patients in Whom the Device Impinges Atrial or Aortic Structures or Protrudes in Superior Vena Cava lumen (SVC)

Impingement	Aortic root	Left atrial wall	Right atrial wall	Protrusion in SVC
	1.1 ± 1 (0 to 3)	0.95 ± 1.6 (-3 to 4)	0.6 ± 1 (-1 to 3)	3.6 ± 4 (0 to 11)
None	-	1	1	7
Positive	11	13	8	13
Contact	9	6	11	-

The degree of contact of the device with the atrial or aortic wall or it’s protrusion in the SVC is given in millimetres (mean ± SD; range).

## Discussion

Chest pain after IASC-C can be related to the procedure itself, for example as a result of device friction or laceration of surrounding cardiac structures like the aortic root or the atrial walls. On the other hand, symptoms may result from, among other causes, concomitant CAD, or be pleuro-pericardial or musculoskeletal. Furthermore, nickel allergy has been suggested as a possible cause of acute or chronic chest pain after IASC-C [5-7]. The degree of proximity of implanted devices to neighbouring cardiac structures and their impact on the clinical course is an issue both insufficiently studied and understood. The experience of a late laceration and perforation of cardiac structure, albeit a rare but probably underreported event, justifies in our opinion the concern and effort involved. Some aspects of this study were quite unexpected. First, the number of examined patients in whom the device was in contact or indenting a cardiac structure was surprising. In 55% of patients, the device was indenting the aortic root, in 65% it was indenting the left atrial wall and in 40% the right atrial wall (Table 3). In 45%, 30% and 55% respectively the device was in contact with but not encroaching on the structure. Of course, it is clear that there is probably a selection bias due to the sole fact that most of the patients presented with chest pain. Patients without chest discomfort and thus not undergoing CT scanning may have no or much less device contact with neighbouring cardiac structures. In fact, the subacute contact and “tissue irritation” may be one of the causes of postinterventional chest pain.

Second, the degree of wall indentation by the device was surprising. If we consider that atrial wall thickness varies between 2-6 mm (with significant variation between the right and left wall thickness in healthy and diseased states, as well as within different regions of the same atrium [8, 9]), it seems understandable that device protrusions of more than 1 mm may be regarded as potentially threatening. Considering an (arbitrary) cut off value, for illustration purposes, of more than a 2 mm indentation by the device as potentially disquieting, then four devices (20%) indented the left atrial, three (15%) the right atrial and two (10%) the aortic wall.

The degree of device contact with atrial or aortic structures does not seem to predict laceration or perforation as occurred in our patient [1]. All episodes, but for the aforementioned, resolved without clinical sequelae. On the other hand, indenting of cardiac structures by the device may be one of the causes of chest pain in the early and subacute phases after device implantation.

### Study limitations

Foremost to be mentioned is the small number of patients examined by CT. It remains to be proven that patients who do not experience chest pain after IASC show a lesser degree of indentation of cardiac structures by the device.
